# COVID-19-associated cerebral microbleeds in the general population

**DOI:** 10.1093/braincomms/fcae127

**Published:** 2024-04-15

**Authors:** Malini V Sagar, Neus R Ferrer, Mostafa Mehdipour Ghazi, Kiril V Klein, Espen Jimenez-Solem, Mads Nielsen, Christina Kruuse

**Affiliations:** Department of Neurology, Copenhagen University Hospital—Herlev and Gentofte, Herlev 2730, Denmark; Department of Computer Science, University of Copenhagen, Copenhagen 2100, Denmark; Department of Computer Science, University of Copenhagen, Copenhagen 2100, Denmark; Department of Computer Science, University of Copenhagen, Copenhagen 2100, Denmark; Department of Clinical Pharmacology, Copenhagen University Hospital—Bispebjerg and Frederiksberg, Copenhagen 2400, Denmark; Department of Computer Science, University of Copenhagen, Copenhagen 2100, Denmark; Department of Neurology, Copenhagen University Hospital—Herlev and Gentofte, Herlev 2730, Denmark; Department of Brain and Spinal Cord Injury, Copenhagen University Hospital—Rigshospitalet, Copenhagen 2600, Denmark

**Keywords:** coronavirus, pandemic, matched controls, neuroimaging, cerebral microhaemorrhage

## Abstract

Cerebral microbleeds are frequent incidental findings on brain MRI and have previously been shown to occur in Coronavirus Disease 2019 (COVID-19) cohorts of critically ill patients. We aimed to determine the risk of having microbleeds on medically indicated brain MRI and compare non-hospitalized COVID-19-infected patients with non-infected controls. In this retrospective case-control study, we included patients over 18 years of age, having an MRI with a susceptibility-weighted sequence, between 1 January 2019 and 1 July 2021. Cases were identified based on a positive reverse transcriptase polymerase chain reaction test for SARS-CoV-2 and matched with three non-exposed controls, based on age, sex, body mass index and comorbidities. The number of cerebral microbleeds on each scan was determined using artificial intelligence. We included 73 cases and 219 matched non-exposed controls. COVID-19 was associated with significantly greater odds of having cerebral microbleeds on MRI [odds ratio 2.66 (1.23–5.76, 95% confidence interval)], increasingly so when patients with dementia and hospitalized patients were excluded. Our findings indicate that cerebral microbleeds may be associated with COVID-19 infections. This finding may add to the pathophysiological considerations of cerebral microbleeds and help explain cases of incidental cerebral microbleeds in patients with previous COVID-19.

## Teaser

Cerebral microbleeds are frequent incidental findings on brain MRI and indicate a risk of brain haemorrhage. Coronavirus Disease 2019 (COVID-19) infections may associate with an increased occurrence of microbleeds, regardless of disease severity. The pathophysiology of these microbleeds is yet to be resolved.

## Graphical Abstract

**Graphical Abstract:**
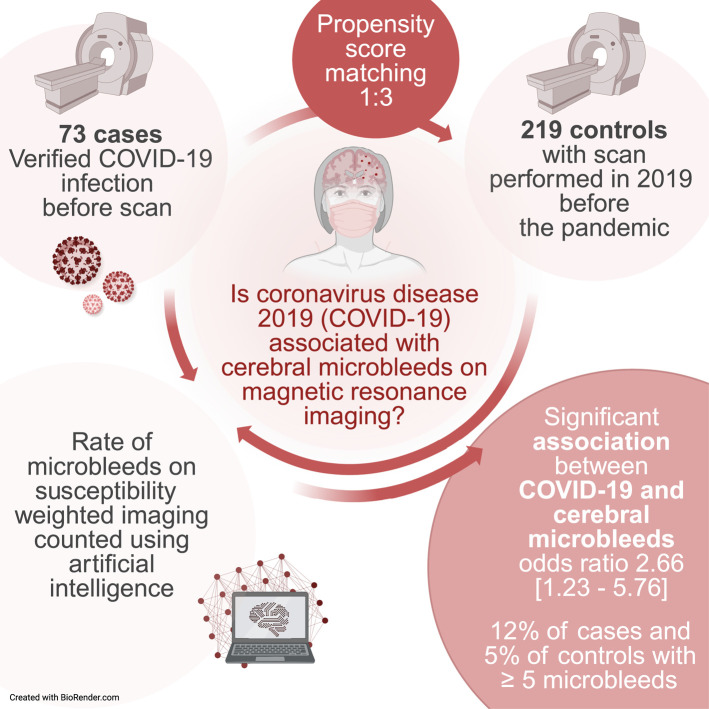

## Introduction

Severe Acute Respiratory Syndrome Coronavirus-2 (SARS-CoV-2) is the highly transmissible pathogen responsible for the Coronavirus Disease 2019 (COVID-19) pandemic.^1^ The severity of the acute phase of COVID-19 varies and ranges from asymptomatic to severe and deadly infection^1^ but may also invade the nervous system^2^ and cause lingering neurologic symptoms.^3^

Cerebral microbleeds (CMB) are frequent incidental findings on MRI and are usually associated with arterial vascular disease, old age and dementia and inform an increased risk of a cerebral haemorrhage.^4^ The clinical consequences of the incidental findings are poorly understood.^4^ From an imaging point of view, COVID-19 has been associated with a multitude of findings, which also include CMB on MRI.^3,5-12^ Most CMB-related studies have focused on critically ill cohorts, without a non-COVID-19 control group,^7-11,13^ leaving it impossible to identify whether CMB are preexisting phenomena, arise due to critical illness, or are a direct consequence of COVID-19 itself.^14^ To date, only one study comprised of a mainly non-hospitalized population, and a non-COVID-19 control group with matched controls exists^15^ where cases and controls had similar numbers of CMB. However, imaging was performed with a primary intent of research, where contact with healthcare services was not a prerequisite, as opposed to being based on medical indication.^15^

CMB in critically ill COVID-19 patients have a predilection for the juxtacortical white matter, internal capsule and splenium of the corpus callosum, compatible with the anatomical distribution of CMB in pre-pandemic patients in intensive care.^16^ The CMB occurring in critically ill COVID-19 patients were hypothesized to be associated with a high central venous pressure of mechanical ventilation or extracorporeal membrane oxygenation (ECMO),^6,16^ hypoxia or therapeutic anticoagulation.^17^ Another proposed mechanism for CMB is the endothelial hypothesis, i.e. angiotensin-converting enzyme 2 receptor (ACE-2) activation by either the virus itself or due to parainfectious cytokine storm.^6^ The corpus callosum, and splenium in particular, has a high concentration of cytokine receptors, rendering it especially susceptible to hyperinflammatory changes.^6^ This aforementioned anatomic distribution of CMB is less likely to occur with other known causes of microbleeds, such as hypertensive arteriopathy or cerebral amyloid angiopathy (CAA).^4,7^

We aimed to determine the risk of CMB in a general COVID-19 population, compared with non-COVID-19 controls in a real-life cohort where MRIs were performed due to medical necessity.

## Materials and methods

### Study design and participants

**Figure 1:**
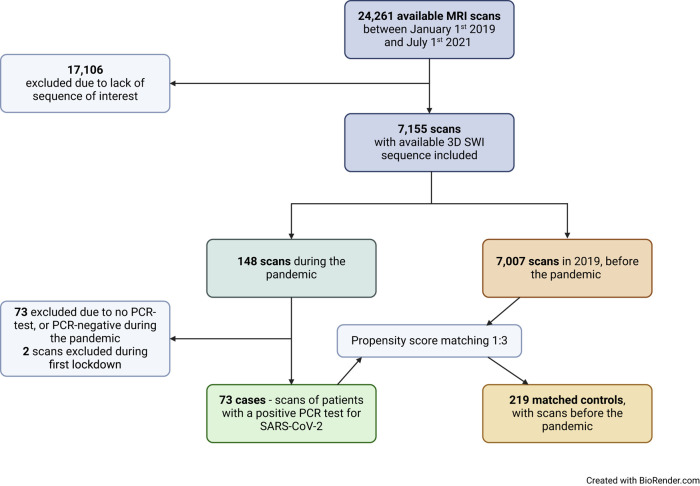
**Data collection.** Flowchart of data retrieval and selection of cases and controls.

Our study was performed retrospectively, where each case was matched with three controls (Fig. 1). Cases were identified based on a positive reverse transcriptase polymerase chain reaction (PCR) (RT-PCR) test and matched with controls based on scan characteristics^18^ (echo time, magnetic field strength and slice thickness and manufacturer), as well as factors available in electronic health records, such as age, sex, body mass index, smoking and the following comorbidities based on International Classification of Diseases 10th Revision (ICD-10) codes and Anatomical Therapeutic Chemical (ATC) codes: diabetes mellitus, ischaemic heart disease, heart failure, cardiac arrhythmia, stroke, bronchial asthma, sleep apnoea, arthritis, osteoporosis, dementia, psychiatric disorders, immunodeficiency, neurologic disorders, cancer, chronic kidney disease, renal dialysis and hypertension.^19^ Anticoagulant use was determined based on ATC codes and included vitamin K antagonists, direct oral anticoagulants, platelet inhibitors, unfractionated heparin and low molecular weight heparin. Field strength was either 1.5 or 3 Tesla, echo time was either 15–25 ms or 35–45 ms, and slice thickness was either 2 or 4 mm. The matching algorithm was a greedy propensity score (PS)-based matching with a caliper width^20^ of 0.2/sigma.^21^ Matching was evaluated by comparison of PS and continuous covariate distribution with the Kolmogorov–Smirnoff test. Dichotomous covariates were tested using Fisher’s exact test on the odds ratios (ORs) or χ^2^ test as appropriate.

Controls had their MRI in 2019, i.e. before the pandemic, eliminating the risk of earlier undiagnosed COVID-19 at the time of the MRI.

Subgroup analysis was carried out for patients with dementia, hospitalized versus non-hospitalized patients, more than versus less than 3 months between a positive RT-PCR test and MRI and for age groups <60 years, 60–70 years and >70 years.

A three-dimensional (3D) deep learning framework was trained and evaluated on 3879 3D scans including in total 38 456 CMB used to evaluate the number, position and size of the microbleeds in the 3D susceptibility-weighted images (SWI) on MRI.^22^

Radiology referrals and reports were reviewed by author M.V.S., a neurology resident, whereby data regarding medical indication, number of microbleeds and presence of tumours was extracted, for comparison.

### Data sources and processing

All patients aged ≥18 who had an MRI performed with an SWI sequence in a hospital in the Capital Region of Denmark, with 1 891 871 inhabitants,^23^ between 1 January 2019 and 1 July 2021, were eligible. Patients with scans during the first Danish lockdown (13 March–17 April 2020) were excluded, since RT-PCR testing was not abundantly available at that time and the help-seeking behaviour of patients was affected by the onset of the pandemic.

### Exposure

Exposure was defined as having a positive RT-PCR test, performed in hospital, either before or up to 3 days after the MRI to ensure a high probability of exposure prior to the scan, where mean incubation times of the COVID-19 variants during the study period varied between 4.43 and 5.18 days.^24^

### Outcome

Our outcome was the presence of ≥5 CMB^25^ of diameter 2–10 mm on SWI.

### Statistical analysis

ORs, their 95% confidence interval and difference from 1 were computed by Fisher’s exact test using a significance level of 0.05.

### Ethics

All methods were carried out in accordance with relevant guidelines and regulations. The study was approved by legal and ethics boards, including the Danish Patient Safety Authority (Styrelsen for Patientsikkerhed, approval #31-1521-257) and the Danish Data Protection Agency (Datatilsynet, approval #P-2020-320).

## Results

### Relationship between positive PCR test for SARS-CoV-2 and CMB on MRI

Out of 7155 eligible scans, we included 73 cases and 219 matched non-exposed controls. Demographics and comorbidities, hence our basis for matching, are presented in Table 1.

**Table 1:** Overview of demographics and comorbidities in cases and controls

	Cases	Controls	Odds ratio (95% CI)	*P*_Fisher_
Age (years)	67.1 **±** 1.7	66.73 **±** 0.02		
BMI	26.3 **±** 0.6	26.7 **±** 0.4		
Female sex, *n* (%)	42 (57)	110 (50)	1.3 (0.8–2.4)	0.34
Diabetes mellitus, *n* (%)	27 (37)	89 (41)	0.9 (0.5–1.5)	0.69
Ischaemic heart disease, *n* (%)	4 (5.5)	22 (10)	0.5 (0.2–1.6)	0.34
Heart failure, *n* (%)	0 (0)	0 (0)	1.0 (0.0 –)	1.00
Cardiac arrhythmia, *n* (%)	5 (6.8)	14 (6.4)	1.1 (0.4–3.1)	1.00
Stroke, *n* (%)	40 (55)	105 (48)	1.3 (0.8–2.2)	0.35
Bronchial asthma, *n* (%)	22 (30)	50 (23)	1.5 (0.8–2.6)	0.21
Sleep apnoea, *n* (%)	0 (0)	0 (0)	1.0 (0.0 –)	1.00
Arthritis, *n* (%)	3 (4.1)	10 (4.6)	0.9 (0.2–3.3)	1.00
Osteoporosis, *n* (%)	8 (11)	17 (7.8)	1.5 (0.6–3.5)	0.47
Dementia, *n* (%)	0 (0)	7 (3.2)	0.0 (0.0 –)	0.20
Psychiatric disorders, *n* (%)	1 (1.4)	5 (2.3)	0.6 (0.1–5.2)	1.00
Immunodeficiency, *n* (%)	0 (0)	0 (0)	1.0 (0.0 –)	1.00
Neurologic disorders, *n* (%)	26 (36)	69 (32)	1.2 (0.7–2.1)	0.56
Cancer, *n* (%)	14 (19)	46 (21)	0.9 (0.5–1.7)	0.87
Chronic kidney disease, *n* (%)	2 (2.7)	3 (1.4)	2.0 (0.3–12.4)	0.60
Renal dialysis, *n* (%)	0 (0)	1 (0.5)	0.0 (0.0 –)	1.00
Hypertension, *n* (%)	51 (70)	161 (74)	0.8 (0.5–1.5)	0.55
Anticoagulation, *n* (%)	43 (59)	154 (70)	0.6 (0.3–1.0)	0.08
Smoker, *n* (%)	41 (56)	116 (53)	1.1 (0.6–1.9)	0.69

Infection with COVID-19 was significantly associated with the occurrence of CMB compared with unexposed controls, also among non-hospitalized subgroups and patients without dementia. In patients with COVID-19 infection, 12% had CMB, whereas CMB were found in 5% of controls (Table 2).

**Table 2:** CMB findings based on AI analysis

Population group	Sample size (*N*)	*N* (%) ≥5 CMB (COVID-19)	*N* (%) ≥5 CMB (control)	Odds ratio (≥5 CMB) (95% CI)	*P*-value (Fisher)
**All**	292	9 (12)	11 (5)	2.66 (1.23–5.76)	**0**.**04**
(*t*_scan_ − *t*_test_) < 3 months	212	7 (13)	11 (7)	2.05 (0.88–4.74)	0.13
(*t*_scan_ − *t*_test_) > 3 months	80	2 (10)	0 (0)		0.06
Age ≤ 60	80	1 (5)	0 (0)		0.25
60 < Age ≤ 70	80	1 (5)	4 (7)	0.74 (0.11–4.85)	0.77
70 < Age	132	7 (21)	7 (7)	3.54 (1.37–9.14)	**0**.**03**
**Non-dementia**					
All	268	9 (13)	10 (5)	3.31 (1.47–7.45)	**0**.**01**
<3 months	188	7 (15)	8 (6)	2.57 (1.07–6.17)	0.07
>3 months	80	2 (10)	0 (0)		0.06
Age ≤ 60	76	1 (5)	0 (0)		0.25
60 < Age ≤ 70	72	1 (6)	4 (7)	0.74 (0.11–4.87)	0.77
70 < Age	120	7 (23)	5 (6)	5.17 (1.84–14.56)	**0**.**01**
Hospitalized	36	1 (11)	2 (7)	1.56 (0.19–12.97)	0.59
Non-hospitalized	232	8 (14)	12 (7)	3.82 (1.57–9.29)	**0**.**01**
**Hospitalized**	40	1 (10)	2 (7)	1.56 (0.19–12.76)	0.59
**Non-hospitalized**					
All	252	8 (13)	9 (5)	2.91 (1.26–6.27)	**0**.**03**
<3 months	176	6 (14)	9 (7)	2.16 (0.86–5.40)	0.14
>3 months	76	2 (11)	0 (0)		0.06
Age ≤ 60	72	1 (6)	0 (0)		0.25
60 < Age ≤ 70	64	1 (7)	3 (7)	1.00 (0.14–7.07)	0.69
70 < Age	116	6 (22)	4 (5)	3.52 (1.27–9.80)	**0**.**04**

Data include subgroups, with OR (with 95% CI) and *P*-value based on Fisher’s exact test. Subgroups were generated by exclusion of entire tetrads of matched cases and controls. Bold values imply statistical significance.

The PS distribution for cases and controls has been compared using the Kolmogorov–Smirnov test with *P* = 0.9999.

### Indications and findings based on referrals and radiology reports

The artificial intelligence (AI)-retrieved CMB matched the radiology reports when these were available (Table 3) in 94% of our included scans. Only a minute number of patients were referred due to COVID-19, or sequelae thereof, whereas the bulk of our scans were carried out due to conventional clinical indications, such as suspected stroke or non-vascular brain disease.

**Table 3:** Brain scan indication and findings retrieved from referrals

	Cases, *n* (%)	Controls, *n* (%)	Total, *n* (%)	*P*-value (χ^2^)
**Radiology report available**	70 (96)	208 (95)	278 (95)	0.99
**Radiologists’ findings**				
Determined if CMB ≥ 5	44 (63)	95 (46)	139 (50)	0.02
Match between AI and radiologist (CMB ≥ 5)	42 (95)	88 (93)	130 (94)	0.80
Radiologist reports tumour	4 (6)	24 (12)	28 (10)	0.24
**Indication for scan**				
Indication stated	70 (96)	207 (95)	277 (95)	0.99
Stroke	54 (77)	137 (66)	191 (69)	0.12
Non-vascular brain disease	16 (23)	68 (33)	84 (30)	0.16
Cognitive symptoms	<5 (6)	13 (6)	17 (6)	0.99
Explicitly COVID-19 related	<5 (4)^a^	0 (0)	<5 (1)	0.02

Percentages among indications add up to more than 100, since a few referrals had multiple simultaneous indications.

^a^Referred due to lingering postinfectious cognitive symptoms or due to decreased level of consciousness after a prolonged stay in the intensive care unit (ICU).

## Discussion

In this retrospective population-based case-control study using AI-based analysis, we found a significantly increased occurrence of CMB in COVID-19 cases compared with matched controls, with CMB in 12% of COVID-19 cases and 5% in controls. The significant difference persisted when excluding patients known to have an increased risk of CMB, i.e. patients with dementia and critical illness. Our results indicate that COVID-19 may be associated with an increased risk of CMB.

Due to our retrospective study design, the risk of confounding cannot be eliminated. Our results may have an inherent bias due to the known overlap between the risk of severe COVID-19 and old age or dementia, the latter independently associated with an increased occurrence of CMB. However, cases and controls were well matched one to three based on scan characteristics, basic demographics and a range of comorbidities, and increased CMB findings persisted when these patients with a high risk of CMB were excluded. High-quality matching was achievable due to a large original cohort of 7155 patients with available SWI sequences, from which cases and controls were selected. Additionally, our included scans were retrieved from a consecutive, i.e. non-selected cohort of patients, and void of self-reported data, hence with low risk of bias. Also, cases were identified based on a positive PCR test, which is highly sensitive.^26^ The AI algorithm had an excellent agreement with radiology reports. Scans with artefacts were not excluded, as the AI algorithm was trained including such data and considered robust. Our included scans were performed in multiple hospitals, hence on different MRI machines, which were also represented in our training data set.^22^ The included scans were solely analysed by our AI algorithm, which provided a monofactorial read on the number of CMB. This may limit the generalizability of our results. Clinical MRIs are primarily interpreted by human radiologists today, where multiple factors contribute to the read. This includes bedside features from referrals, concurrent findings on other sequences than SWI and flexibility with regard to MRI settings, which our AI algorithm did not take into account. On the contrary, this one-track aspect of our AI algorithm may also reduce bias in the interpretation process, where risks of inter- and intrarater variability are potentially reduced. A comparison of our AI algorithm and clinical radiology is of interest, but out of scope with regard to this study. However, MRI features that associate with an increased risk of CMB, such as signs of small vessel disease, would have been of interest. Especially considering potential aetiologies of these COVID-19 associated CMB. Nevertheless, we have taken risk factors of small vessel disease such as diabetes mellitus, hypertension, age and BMI^27^ into account, in our matching process. An important confounder could be the influence of the pandemic on decision-making by both patients and professionals, i.e. differences in healthcare-seeking behaviour. Due to fear of either infection or burdening the healthcare system, patients who were scanned during the pandemic may have, on average, been more ill. This may not fully be represented in the matching process, based on ICD-10 codes. Information on patients hospitalized with COVID-19 was not a part of our matching process and could be a potential confounding factor. We attempted to alleviate this by creating a non-hospitalized subgroup, where the increased rate of CMB persists among COVID-19 patients. Apart from hospitalization, we did not have data regarding potential complications of COVID-19, which would have been of interest, and could have shed light on potential causality behind the increased rate of microbleeds.

Further limitations were a limited insight into the clinical characteristics of our included patients; hence, it is currently undetermined, whether CMB after COVID-19 have a syndromic correlate or not. In the case of the former, our results may guide the investigation and treatment of patients suffering from e.g. lingering postinfectious symptoms. Contrarily, if asymptomatic and clinically inconsequential, our results will aid the interpretability of the incidental yet relatively frequent occurrence of CMB on MRI. During the matching process, we did not account for grounds of referral to MRI, which was a potential source of bias. However, indications of scans were similar among cases and controls, except for indications that were related to COVID-19 (Table 3).

Our cut-off value of minimum five CMB per scan was chosen based on the potential clinical consequences thereof. Among patients with previous ischaemic stroke with less than five CMB, the risk of recurrent stroke has been shown to be greater than the risk of intracerebral haemorrhage (ICH).^25^ However, if five or more CMB are present, these risks are comparable^25^ where difficult clinical decisions with regard to antiplatelet or anticoagulant therapy arise, albeit this cut-off implies a risk of false negative scans in our data set. To mitigate this issue, we carried out a *post hoc* sensitivity analysis, where cut-off values between 3 and 18 CMB were used, where the rate of CMB among the COVID-19 cases remained greater than among the controls.

Although the pathophysiology of these CMB remains undetermined, our findings could add to the understanding of both CMB- and COVID-19 related neurological consequences. The occurrence in non-hospitalized COVID-19 patients indicates that CMB are not solely attributable to critical illness and treatment thereof. Hence, our study may support an endothelial hypothesis related to CMB in COVID-19. A single study of encephalopathic COVID-19 patients who underwent MRI found that patients with arterial enhancement on black-blood sequences also had increased numbers of CMB,^8^ and endotheliitis associated with increased expression of ACE2 receptors has been demonstrated upon autopsies of COVID-19 patients.^27^

Incidental occurrences of CMB on MRI are often attributable to or indicative of CAA, neurodegenerative disease or hypertensive arteriopathy.^4^ Since CMB due to these chronic conditions are associated with an increased risk of ICH,^28^ their occurrence influences therapeutic decision-making, causing relevant restraint among clinicians, regarding the use of e.g. anticoagulants,^28^ as well as newer treatments for Alzheimer’s disease of which ICH is a potential side-effect.^29^ The risk of ICH, in the case of CMB after COVID-19, is not known. However, potentially excluding patients with postinfectious CMB from these important treatment options may be unwarranted, especially if the risk of additional CMB fades postinfection. On the same note, our study underlines the relevance of clinical symptoms of CAA to be included in the often highly consequential diagnosis of probable CAA, as is the case in the updated Boston 2.0 criteria.^30^

Regardless of severity, COVID-19 is known for its risk of long-term neurologic symptoms. It is yet to be determined, whether CMB in patients who have had COVID-19 have corresponding clinical neurologic manifestations. A glimpse at the matter is provided via our access to MRI referrals, which, apart from a low number of cases, were indicated based on neurological or other conditions unrelated to COVID-19, e.g. suspected stroke.

Petersen *et al.*^15^ previously showed that non-hospitalized COVID-19 patients had incidental CMB findings comparable with matched healthy controls. MRI scans in this study were carried out in an otherwise healthy cohort as part of a research protocol. However, our setting and population was different, as we only included scans carried out due to real-life medical necessity, which to a greater degree is representative of actual patients. Hence, our results provide an alternate, yet clinically applicable, perspective. The discrepancy in results between the two studies may be indicative of the differences between the two populations, from which study patients originated, i.e. the occurrence of CMB after COVID-19 may to some degree be associated with comorbidities and neurologic symptoms.

Interpretation of incidental findings is a gruelling task of clinical practice, and CMB on brain MRI are classical examples^31^ of where there is still uncertainty on the clinical implications and actions required.^4^ If some incidental CMB can be attributed to COVID-19, this task becomes less of a challenge.

However, further investigation into the corresponding clinical features of these patients is warranted.

In conclusion, COVID-19 may increase the risk of CMB also in patients without dementia and in the non-critically ill population. Our results indicate a separate cause of CMB, other than those related to chronic conditions, such as dementia, CAA or hypertensive arteriopathy, which potentially carry therapeutic consequences, e.g. avoidance of anticoagulants. Hence, our study indicates that alternate causes of CMB should be accounted for, during clinical decision-making, when relevant.

## Data Availability

Due to confidentiality constraints, patient data from our study cannot be made available.
